# Quaternary structure predictions of transmembrane proteins starting from the monomer: a docking-based approach

**DOI:** 10.1186/1471-2105-7-340

**Published:** 2006-07-12

**Authors:** D Casciari, M Seeber, F Fanelli

**Affiliations:** 1 Department of Chemistry, Dulbecco Telethon Institute (DTI), University of Modena e Reggio Emilia, Via Campi 183, 41100 Modena, Italy

## Abstract

**Background:**

We introduce a computational protocol for effective predictions of the supramolecular organization of integral transmembrane proteins, starting from the monomer. Despite the demonstrated constitutive and functional importance of supramolecular assemblies of transmembrane subunits or proteins, effective tools for structure predictions of such assemblies are still lacking. Our computational approach consists in rigid-body docking samplings, starting from the docking of two identical copies of a given monomer. Each docking run is followed by membrane topology filtering and cluster analysis. Prediction of the native oligomer is therefore accomplished by a number of progressive growing steps, each made of one docking run, filtering and cluster analysis. With this approach, knowledge about the oligomerization status of the protein is required neither for improving sampling nor for the filtering step. Furthermore, there are no size-limitations in the systems under study, which are not limited to the transmembrane domains but include also the water-soluble portions.

**Results:**

Benchmarks of the approach were done on ten homo-oligomeric membrane proteins with known quaternary structure. For all these systems, predictions led to native-like quaternary structures, i.e. with C_α_-RMSDs lower than 2.5 Å from the native oligomer, regardless of the resolution of the structural models.

**Conclusion:**

Collectively, the results of this study emphasize the effectiveness of the prediction protocol that will be extensively challenged in quaternary structure predictions of other integral membrane proteins.

## Background

A number of α-helical transmembrane (TM) proteins organize themselves in supramolecular assemblies, which constitute the functional units (i.e. biological units) of ion/water channels, electron or proton transfer proteins as well as transporters [1]. Also the 7-TM bacteriorhodopsin (BRD) forms trimers, which, in turn, form multimolecular assemblies in the native purple membrane [2,3]. G-protein-coupled receptors (GPCRs), which constitute the largest superfamily of membrane proteins, are not exceptions to this rule. Although they have been classically assumed to exist and function as monomeric entities, the concept that they exist as constitutive dimers/oligomers is now substantiated by ever increasing evidences from *in vitro *studies (reviewed in refs. [4-7]). Predictions of the likely interfaces in GPCR dimers done so far essentially relied on sequence-based methods [8-13] (reviewed also in Ref[14]).

Despite the demonstrated constitutive and functional importance of supramolecular assemblies of TM subunits or proteins, effective tools other than sequence-based methods for structure predictions of such assemblies are still lacking. Indeed, almost all the docking algorithms and approaches to quaternary structure predictions developed so far work with water-soluble proteins and most of them employ geometrical constraints, including symmetry information [15-20]. The very few approaches intended for quaternary structure predictions of TM-helical proteins include a method based on Monte Carlo sampling of single TM helices [21]. The method is limited to very simple homo-oligomers and requires knowledge about the oligomerization state of the protein, since symmetry information is used to filter the most realistic solutions. The information from cryo-electron microscopy and evolutionary data is employed by an alternative automated method for orienting TM helices [22]. The method has been probed on a number of membrane proteins, including the multimeric TM portion of the acetylcholine receptor [22]. Another docking method, i.e. GRAMM [23], can deal with predictions of helix-helix packing interactions involving either water-soluble or TM-systems. In fact, GRAMM can run in a "helix docking mode" that discards configurations with large displacements along the helix axes and angles between helices larger than indicated cutoffs [24]. This is instrumental in saving computational time and facilitating the analysis of the docking results.

Herein, we present an approach based upon rigid body docking simulations, membrane topology-based filtering, and cluster analysis to predict the quaternary structure of homo-oligomeric integral membrane proteins. This approach does not employ symmetry constraints either for improving sampling or in the filtering step. Furthermore, there are no size-limitations in the systems under study, which are not limited to the TM domains but include also the water-soluble portions.

Benchmarks of the approach were first carried out on the tetrameric KcsA potassium channel H^+ ^gated (384 amino acids, PDB code: 1BL8; Figure 2) [25], pentameric MscL and eptameric MscS mechanosensitive channels (540 amino acids, PDB code: 1MSL, Figure 3[26], and 1771 amino acids, PDB code: 1MXM, Figure 4) [27], respectively), and trimeric BRD (698 amino acids, PDB code: 1BRR; Figure 5) [2]. Selection of these supramolecular systems followed an accurate inspection of the database of Membrane Protein structures from White's laboratory [1]. The selected proteins, indeed, fulfilled at best the following requirements: (a) the biological unit is a homo-oligomer; (b) the asymmetric crystallographic unit contains the biological unit; (c) the monomers in the biological unit are α-helical TM proteins; and (d) significant structural diversity exists among the selected oligomers concerning oligomeric order, architecture and extension of the intracellular and extracellular domains. Successively, benchmarks have been extended to a number of homo-oligomeric transmembrane proteins, selected from the same database, for which the asymmetric unit is constituted by the monomer. They include a dimer, i.e. the membrane spanning region of the BtuCD Vitamin B12 Transporter (648 amino acids, PDB code: 1L7V) [28]; trimers like the AmtB ammonia channel (1146 amino acids, PDB code: 1U77) [29] and the AcrB bacterial multi-drug efflux transporter (3108 amino acids, PDB code: 1IWG); and tetramers like the AQP1 aquaporin water channel (996 amino acids, PDB code: 1J4N) [30], the GlpF glycerol facilitator channel (1016 amino acids, PDB code: 1FX8) [31], and the KirBac1 Inward-Rectifier Potassium channel (1032 amino acids, PDB code: 1P7B) [32].

**Figure 1:**
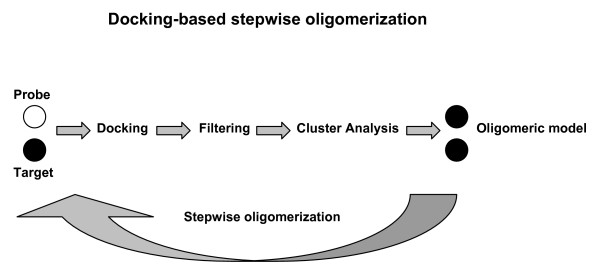
Flowchart of the docking-based stepwise oligomerization approach. The quaternary structure prediction approach consists in a number of dense docking samplings, starting from the docking of two identical copies of a given monomer. Each docking run is followed by membrane topology filtering and cluster analysis. Thus, prediction of the native oligomer is accomplished by a number of progressive growing steps, each made of one docking run, filtering and cluster analysis. For each stepwise quaternary structure prediction, the docking runs that succeeded the first one were carried out by using the original monomer as a probe and the intermediate oligomer as a target.

**Figure 2:**
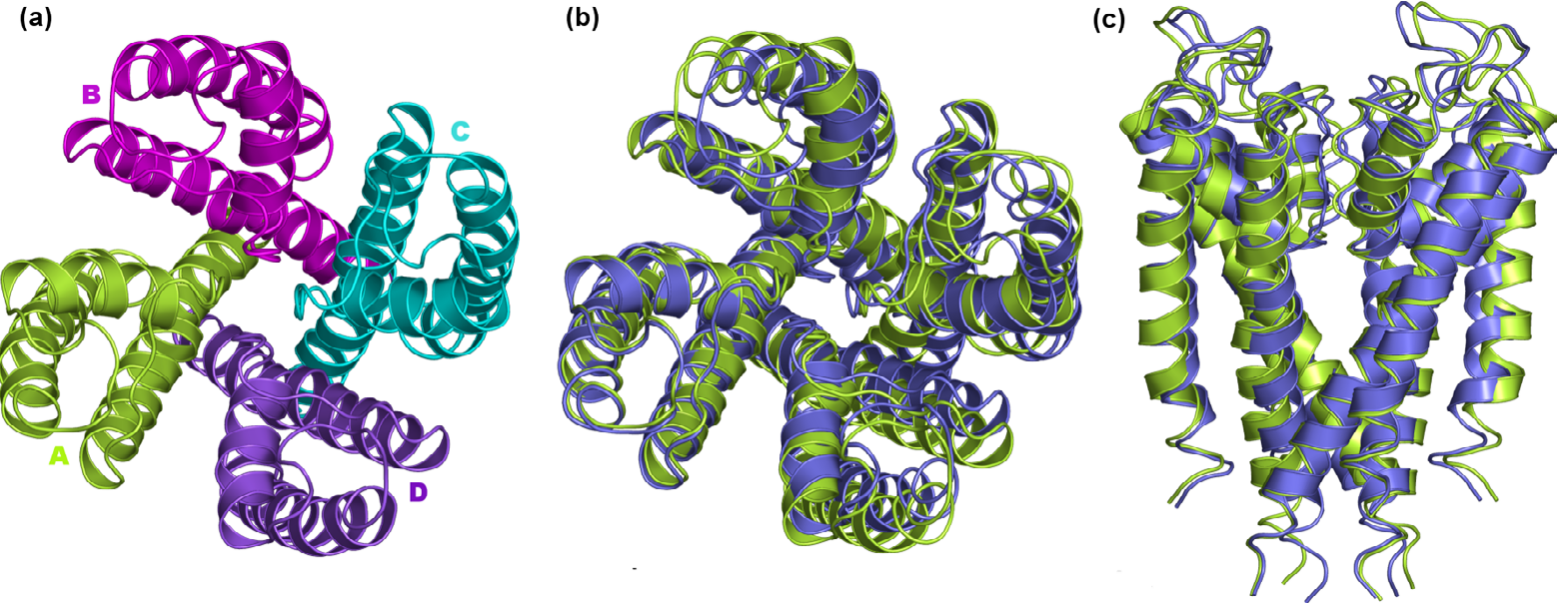
Native and native-like structures of tetrameric KcsA. (a) View of the crystal structure seen from the extracellular side; the monomers are differently colored. (b) and (c) The superimposition between native (green color) and the best native-like (violet color) structures is shown. The native-like structure shown in this figure, i.e. the A-Bs1-Cs500-Ds2 tetramer, has been achieved through a dipole moment-based reorientation approach (see Table 1 and Figure 6). In panel (b) the superimposed structures are seen from the extracellular side, whereas in panel (c) the structures are seen in a direction parallel to the membrane surface. Drawings were done by means of the software PYMOL 0.98 [39].

**Figure 3:**
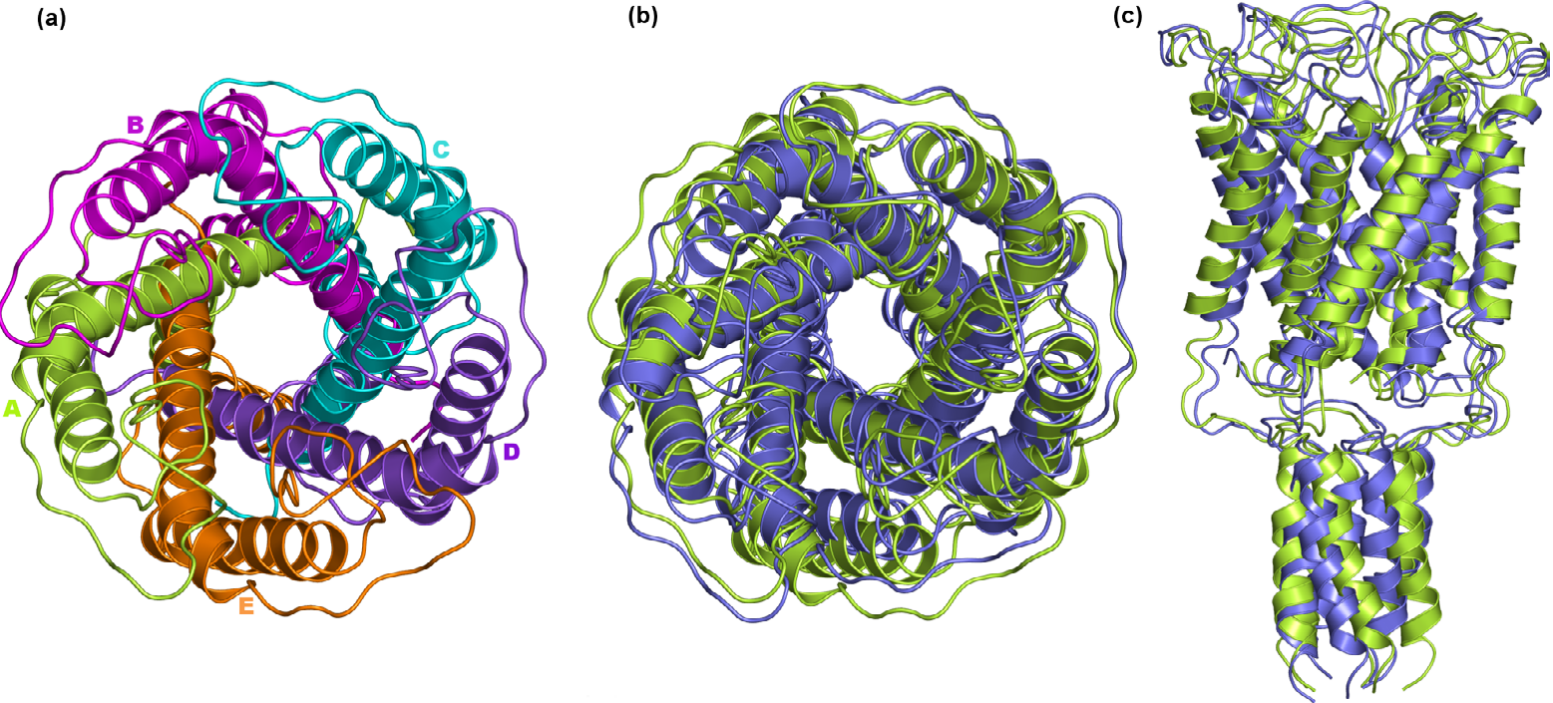
Native and best native-like structures of pentameric MscL. The best predicted native-like pentamer (violet color) is the one encoded as A-Bs8-Cs1-Ds1-Es6 (see Table 1 and Figure 7). The description of this figure is like that of Figure 2.

**Figure 4:**
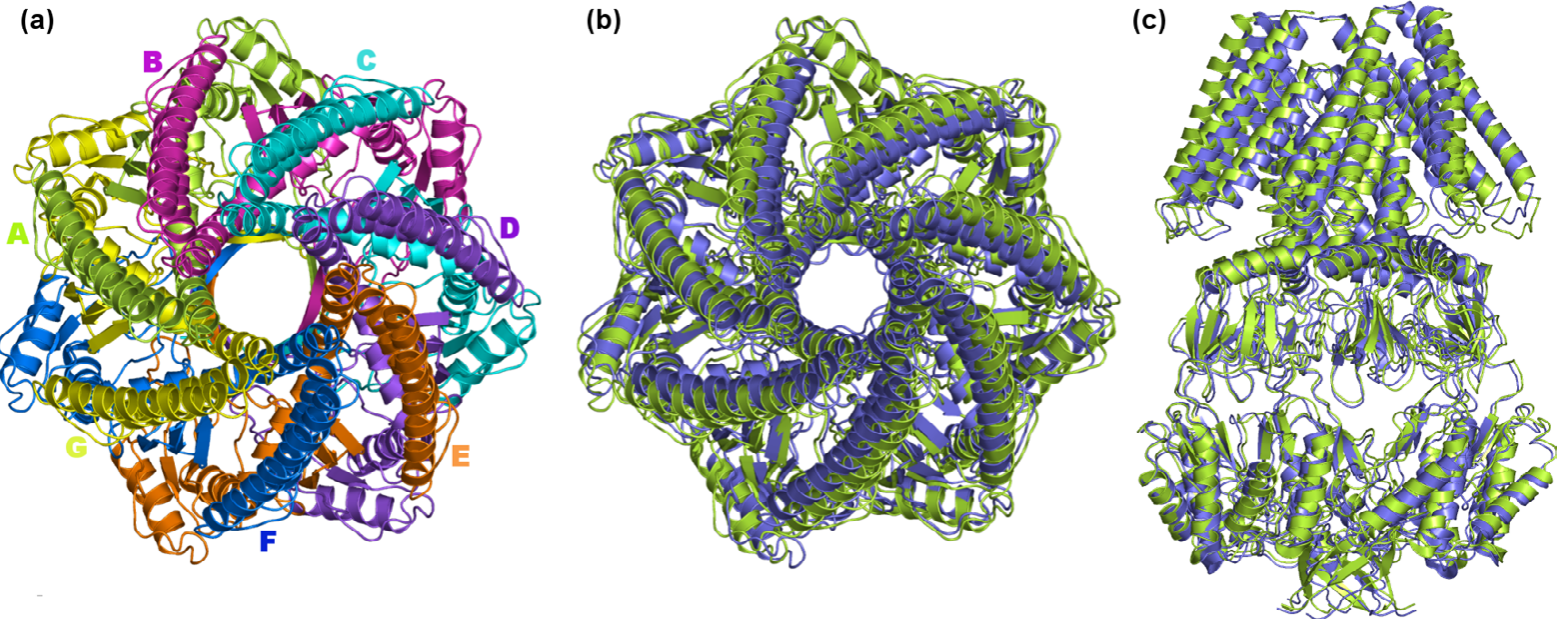
Native and best native-like structures of eptameric MscS. The best predicted native-like eptamer (violet color) is the one encoded as A-Bs3-Cs1-Ds1-Es6-Fs1-Gs1 (see Table 1 and Figure 8). The description of this figure is like that of Figure 2.

**Figure 5:**
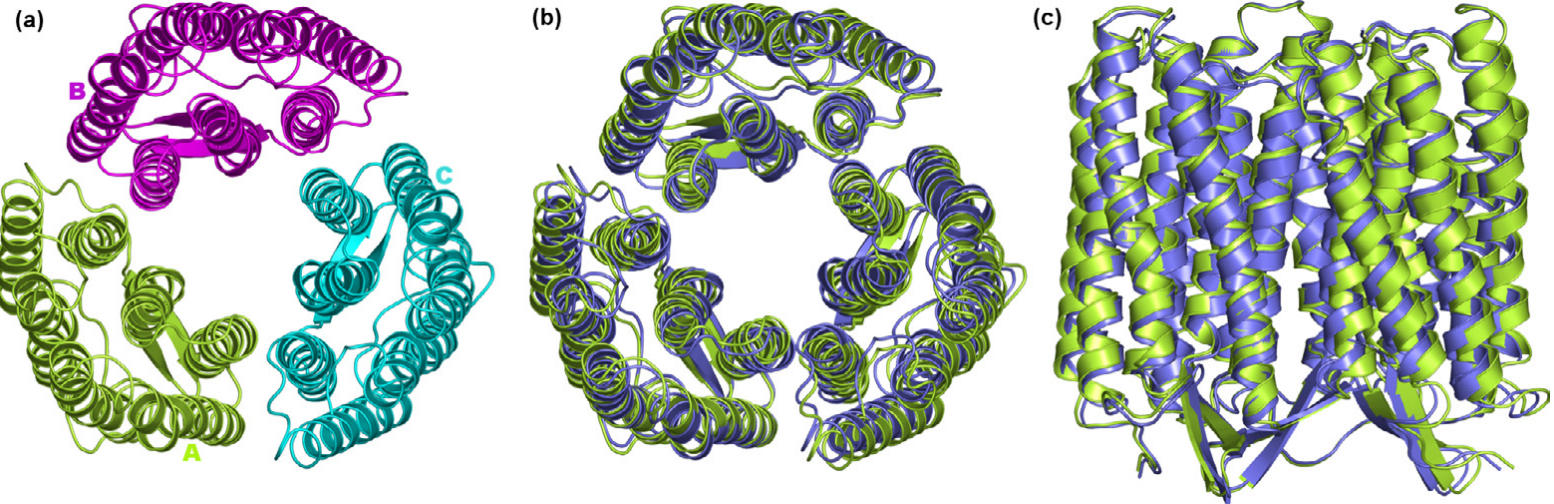
Native and native-like structures of trimeric BRD. The predicted native-like eptamer (violet color) is the one encoded as A-Bs162-Cs14 (see Table 1 and Figure 9). The description of this figure is like that of Figure 2.

The structure of the biological unit from the PDB, obtained by symmetry operations, was used as a native complex for each of these proteins.

For all the systems under study, the approach led to native-like quaternary structures, i.e. with a Root Mean Square Deviation of the C_α_-atoms (Cα-RMSD) lower than 2.5 Å from the native oligomer, regardless of the resolution of the structural models.

The effectiveness of the approach makes it suitable for predictions of the supramolecular architecture of other integral membrane proteins.

## Results

The computational approach developed in this study consists of a number of dense docking samplings, starting from the docking of two identical copies of a given monomer. Each docking run is followed by membrane topology filtering and cluster analysis. Thus, prediction of the native oligomer is accomplished by a number of progressive growing steps, each made of one docking run, filtering and cluster analysis (schematized in Figure 1)).

The only requirement with this approach is the structural model of the monomer and the knowledge of a set of C_α_-atoms, which lie at the two lipid/water interfaces, defining two parallel planes. In the target monomer, these two planes must be parallel to the xy plane and, hence, perpendicular to the z-axis. If these planes are parallel to the xy plane and, hence, perpendicular to the z-axis, the orientation of the monomer is considered good and no reorientation is needed. In contrast, if such planes are not parallel to the xy plane, the monomer needs a reorientation. This reorientation is done by computing the rotations necessary to bring the two planes parallel to the xy plane and then using an average between the two rotations to reorient the whole monomer. The correct membrane topology of the target monomer is, indeed, necessary for the membrane topology filter to work properly (see Methods).

The following paragraphs summarize the results of benchmarks done on ten selected oligomers.

### Quaternary structure predictions of oligomeric channels: KcsA, MscL and MscS

The A subunit of KcsA is constituted by 96 amino acids (i.e. 23–119 sequence) organized in two TM α-helices connected by the 30 amino acid pore region [25]. The A subunit extracted from the crystal structure was used both as a target and a probe in the first step, and as a probe in the following docking simulations. Prior to the first docking run, a reorientation of the KcsA A subunit was needed to put it in the right orientation with respect to the putative membrane. Two different ways for reorienting the subunit were probed, i.e. a dipole moment-based approach and a membrane topology-based approach. The first consisted in rotating the native oligomer so as to put the dipole moment of the tetramer parallel to the z-axis and then extracting the A subunit. The second consisted in the following steps: (a) prediction of two sets of C_α_-atoms of the monomer, which lie at the two membrane-water interfaces; (b) determination of the planes that fit at best these sets of atoms; and (c) orientation of these planes parallel to the xy plane. The first reorientation approach, applied to the native oligomer, was instrumental in designing and benchmarking the prediction protocol, whereas the second approach, applied to the monomer, is instrumental in blind predictions.

**Figure 6:**
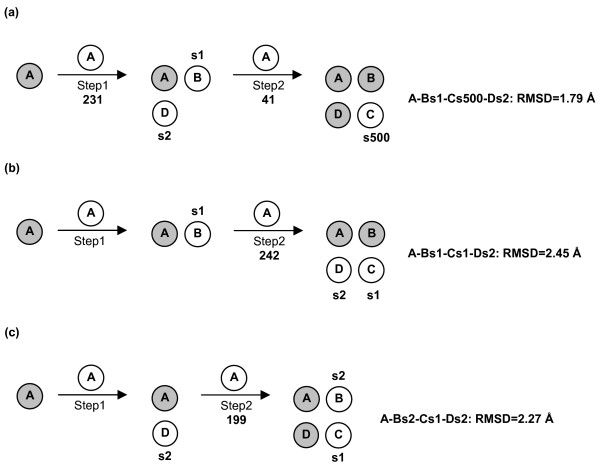
Prediction paths for KcsA. Each of the three different growing paths ((a), (b) and (c)) is characterized by selection, at each growing step, of the best scored solution within the most populated cluster/s, characterized also by similar and significantly low MemTop index. The number of solutions filtered at each step is reported under the arrow. The circle on the arrow indicates the probe, whereas the circles that precede the arrow are the targets. The monomers that constitute these targets are indicated by gray circles except for the last added monomer/s, which are indicated by white circle/s and by the solution number in the ZDOCK output list. The final oligomer is indicated by a string of letters and characters in a way that each subunit is associated with the docking solution. In detail, the upper case letter indicates the subunit, whereas the letter "s" followed by a number indicates the solution number in the ZDOCK output list. Finally, the C_α_-RMSD (Å) between native and predicted quaternary structures is also reported. All the amino acid residues have been included in C_α_-RMSD calculations.

Quaternary structure predictions of KcsA consisted of two alternative two-step growing paths differing for the fact that one employed a trimer (Figure 6a), whereas the other employed a dimer (Figure 6b and 6c) as a target at step 2. The two different paths shared in common step 1, i.e. A vs A docking. A vs A docking gave 231 realistic solutions, i.e. those solutions, which passed the membrane topology filter. These solutions essentially grouped into two clusters, which also showed similar indices of membrane topology goodness (i.e. MemTop indices of 0.495 and 0.494; see the Methods section for the MemTop definition). The best scored solutions from each of these two clusters, s2 and s1 (the letter "s" followed by a number indicates the solution number, or rank number, in the ZDOCK output list), were selected for the following growing step. These solutions contributed to the growth of a cyclic oligomer, likely to represent subunits D and B, respectively, i.e. labeled in a clockwise manner as seen from the extracellular side (Figure 6a). As for the path passing through an intermediate trimer, A vs A-Bs1-Ds2 (the upper case letter indicates the subunit) docking led to 41 realistic solutions, which essentially grouped in four clusters. The best scored solution from the third cluster (characterized by the best MemTop score: 0.375), s500, was selected to produce the A-Bs1-Cs500-Ds2 tetramer. Such tetramer was characterized by a C_α_-RMSD of 1.79 Å from the native structure (Figures 2b, 2c and 6a, Table 1).

**Table 1:** Summary of the benchmark results.

**NAME**^a^	**PDB**^b^	**Res.^c ^(Å)**	**ORDER**^d^	**PATH**^e^	**Pred. Structures**^f^	**C_α_-RMSD**^i^
BtuCD	1L7V	3.2	Dimer	a	**A-Bs1678**^g^	**0.63**
BRD*	1BRR	2.9	Trimer	**b**	**A-Bs162-Cs14**	**1.04**
AmtB	1U77	1.35	Trimer	a	A-Bs7-Cs1	1.17
				b	A-Bs7-Cs1	0.83
				**c**	**A-Bs1-Cs1**	**0.75**
AcrB	1IWG	3.5	Trimer	a	A-Bs4-Cs1	2.78
				**b**	**A-Bs4-Cs1**	**1.56**
				c	A-Bs1-Cs1	1.64
KcsA*	1BL8	3.3	Tetramer	**a**	**A-Bs1-Cs500-Ds2**^h^	**1.79**
				b	A-Bs1-Cs1-Ds2	2.45
				c	A-Bs2-Cs1-Ds2	2.77
AQP1	1J4N	2.2	Tetramer	**a**	**A-Bs2-Cs1-Ds1**	**1.31**
				b	A-Bs1-Cs2-Ds1	1.71
				c	A-Bs2-Cs4-Ds1	1.37
GlpF	1FX8	2.2	Tetramer	**a**	**A-Bs2-Cs1-Ds1**	**1.11**
				b	A-Bs1-Cs2-Ds1	1.16
				c	A-Bs2-Cs2-Ds1	1.39
KirBac1	1P7B	3.65	Tetramer	a	A-Bs3-Cs1-Ds1	1.53
				**b**	**A-Bs3-Cs1-Ds3**	**1.47**
				c	A-Bs2-Cs1-Ds1	1.80
MscL*	1MSL	3.5	Pentamer	a	A-Bs5-Cs1-Ds4-Es6	3.45
				b	A-Bs5-Cs3-Ds2-Es1	2.83
				**c**	**A-Bs8-Cs1-Ds1-Es6**	**2.47**
MscS*	1MXM	3.9	Eptamer	a	A-Bs3-Cs3-Ds3-Es1-Fs1-Gs1	2.21
				b	A-Bs3-Cs3-Ds1-Es165-Fs3-Gs1	11.30
				**c**	**A-Bs3-Cs1-Ds1-Es6-Fs1-Gs1**	**2.06**

^a^Abbreviated name of the membrane proteins subjected to the benchmarks. Asterisks indicate the systems, for which the asymmetric crystallographic unit contains the biological unit.

^b^PDB code of the membrane proteins subjected to the benchmarks.

^c^Atomic resolution (Å) of the crystallographic structures.

^d^Oligomeric order of the biological units. ^e^Probed growing paths (see the text and Figures 6-9 for a detailed explanation). For each protein system, the path that produced the best oligomer, in terms of C_α_-RMSD from the native assembly, is highlighted in bold.

^f^Predicted oligomers: the upper case letter indicates the subunit, whereas the letter "s" followed by a number indicates the solution number in the ZDOCK output list. The best oligomer, in terms of C_α_-RMSD from the native assembly, is highlighted in bold.

^g^The predicted BtuCD dimer was achieved following a dipole-moment-based approach for A subunit reorientation. The same path, but using a membrane topology-based approach for A subunit reorientation, led to the A-Bs1584 dimer characterized by a C_α_-RMSD of 0.48 Å from the native dimer.

^h^The best predicted KcsA tetramer was achieved following a dipole-moment-based approach for A subunit reorientation. The same path, but using a membrane topology-based approach for A subunit reorientation, led to the A-Bs2-Cs12-Ds4 tetramer characterized by a C_α_-RMSD of 0.94 Å from the native oligomer.

^i^C_α_-RMSD (Å) between the native and the predicted oligomer from each growing path. For each system, the lowest C_α_-RMSD is highlighted in bold.

The alternative path passing through an intermediate dimer consisted of two parallel ways, depending on whether the A-Bs1 or the A-Ds2 dimer was employed as a target (Figure 6b and 6c, respectively). In the first case, 242 realistic solutions were filtered, which essentially grouped in two clusters. The best solutions from each of these two clusters, s2 and s1, were, respectively, characterized by contacts with the A and Bs1 monomers in the target dimer, leading to the A-Bs1-Cs1-Ds2 tetramer, which showed a C_α_-RMSD of 2.45 Å from the native structure (Figure 6b, Table 1). The parallel pathway, which used the A-Ds2 dimer as a target at step 2, led to 199 realistic solutions, which essentially grouped in two clusters characterized also by similar MemTop indices (i.e. 0.567 and 0.490). The best solutions from each of these two clusters, s2 and s1, led to the A-Bs2-Cs1-Ds2 tetramer, showing a C_α_-RMSD of 2.27 Å from the native structure (Figure 6c, Table 1).

In summary, the three different growing paths shared in common the first step, i.e. A vs A docking, and all led to native-like tetramers (Figure 6). Among these tetramers, the one obtained by employing a trimer as a target at step 2 showed the lowest C_α_-RMSD from the native complex (i.e. 1.79 Å, Table 1 and Figures 2b, 2c and 2a). The intermediate and final oligomers, with only one exception, were made of solutions falling amongst the top four out of 4000 in the respective output list (Figure 6). The results presented above were achieved by employing a dipole moment-based criterion for the initial reorientation of the target A subunit. However, employing a membrane topology-based approach for subunit reorientation equally led to native-like solutions. In fact, the path employing a trimer as a target at step 2 led to the A-Bs2-Cs12-Ds4 tetramer, showing a C_α_-RMSD of 0.94 Å from the native structure, whereas one of the two paths employing a dimer as a target at step 2 led to the A-Bs1-Cs52-Ds4 tetramer, showing a C_α_-RMSD of 1.94 Å from the native structure. Also in this case, employing an intermediate trimer as a target at step 2 led to better predictions than employing a dimer.

The same general approaches were employed for quaternary structure predictions of the the pentameric MscL and eptameric MscS mechanosensitive channels, but neither one of the two systems required an initial reorientation of the monomer. In detail, the A subunit of the MscL is constituted by 108 amino acids (i.e. 10–118 sequence), organized in two TM α-helices and a third cytoplasmic helix (Figure 3) [26]. The A subunit of MscS is constituted by 253 amino acids (i.e. 27–280 sequence), organized in three TM α-helices and a huge cytosolic domain (i.e. 113–280 sequence, Figure 4) [27].

**Figure 7:**
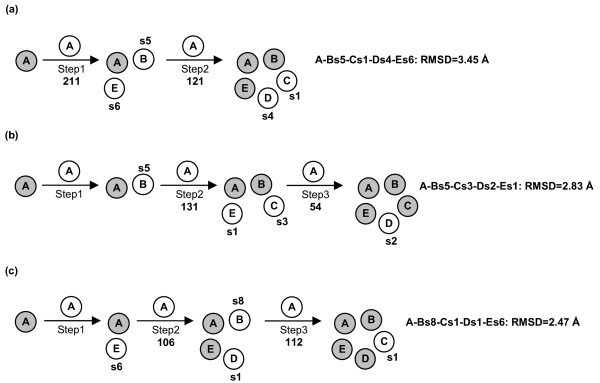
Prediction paths for MscL. See Figure 6 for the description of this figure.

For MscL, two-step and three-step growing paths were probed, differing for the fact that the first path utilized a trimer (Figure 7a), whereas the second one utilized a dimer (Figure 7b and 7c) as a target at step 2. Similarly to the KcsA, the two different growing paths shared in common the first step, i.e. A vs A docking. Thus, A vs A docking gave 211 realistic solutions, essentially grouped in two clusters, showing also similar MemTop indices (i.e. 0.527 and 0.588). As for the two-step growing path, the first run was instrumental in obtaining the A-Bs5-Es6 trimer by employing, simultaneously, the best scored solutions from the first two most populated clusters, s5 and s6 (Figure 7a, bold labels). A vs A-Bs5-Es6 docking led to 121 realistic solutions, which essentially grouped in the first two clusters. The best scored solutions from each of these clusters, s1 and s4, were characterized by contacts with the Bs5 and Es6 monomers, respectively, closing the oligomeric cycle. The A-Bs5-Cs1-Ds4-Es6 pentamer showed a C_α_-RMSD of 3.45 Å from the native structure (Figure 7a, Table 1).

As for the three-step paths, the best scored solutions from the two most populated clusters, s5 and s6, were used to produce the A-Bs5 and A-Es6 dimers. Each of these two dimers was used in turn as a target in two parallel sets of docking simulations, which finally led to the A-Bs5-Cs3-Ds2-Es1 and A-Bs8-Cs1-Ds1-Es6 pentamers, characterized by almost comparable C_α_-RMSDs, i.e. 2.83 Å and 2.47 Å, respectively, from the native oligomer (Figure 7b and 7c, Table 1). Thus, employing a dimer as a target at step 2 was better than using a trimer (Figure 7). Also, all the predicted pentamers were made of solutions falling amongst the top ten out of 4000 in the respective output list (Figure 7, Table 1).

**Figure 8:**
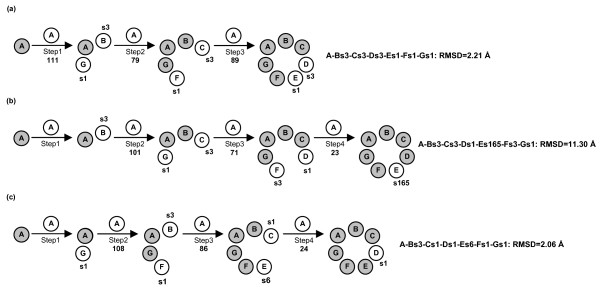
Prediction paths for MscS. See Figure 6 for the description of this figure.

For quaternary structure predictions of MscS, three-step and four-step growing paths were probed, differing for the fact that the first utilized a trimer (Figure 8a), whereas the second utilized a dimer (Figure 8b and 8c) as a target at step 2. Also in this case, the two different growing paths shared in common the first step, i.e. A vs A docking. Thus, A vs A docking gave 111 realistic solutions, essentially grouped in two clusters showing also significantly low MemTop indices (i.e. 0.258 and 0.312).

As for the three-step growing path, the first run was instrumental in obtaining the A-Bs3-Gs1 trimer by using the best scored solutions from the first two most populated clusters, s3 and s1 (Figure 8a). A vs A-Bs3-Gs1 docking led to 79 realistic solutions, which essentially grouped in the first two clusters. The best scored solutions from these clusters, s1 and s3, were, respectively, characterized by contacts with the Gs1 and Bs3 monomers, likely to represent the F and C subunits. A vs A-Bs3-Cs3-Fs1-Gs1 docking produced 89 solutions essentially grouped in the first two clusters, showing also similar MemTop indices (i.e. 0.431 and 0.379). The best scored solution from each of these clusters, s1 and s3, were, respectively, characterized by contacts with the Fs1 and Cs3 monomers and completed the oligomeric cycle, likely to be the E and D subunits, respectively. The thereof obtained A-Bs3-Cs3-Ds3-Es1-Fs1-Gs1 eptamer showed a C_α_-RMSD of 2.21 Å from the native oligomer (Figure 8a, Table 1).

As for the four-step paths, the best scored solutions from the first two most populated clusters, s3 and s1, were used to produce the A-Bs3 and A-Gs1 dimers. These dimers were used in turn as a target in two parallel sets of docking simulations, which finally led to the A-Bs3-Cs3-Ds1-Es165-Fs3-Gs1 and A-Bs3-Cs1-Ds1-Es6-Fs1-Gs1 eptamers, characterized by C_α_-RMSD of 11.30 Å and 2.06 Å, respectively, from the native oligomer (Figure 8b and 8c, Table 1). In summary, the solutions constituting the three alternative eptamers, which were obtained following different paths, fell amongst the top ten out of 4000 in the respective output list (Table 1 and Figure 8). Also, one of the two four-step growing paths produced the eptamer with the lowest C_α_-RMSD from the native oligomer (i.e. 2.06 Å, Table 1 and 4b, 4c and 8c), whereas the other four-step growing path gave the worst eptamer in terms of C_α_-RMSD (i.e. 11.30 Å, Figure 8b). Since the two predicted quaternary structures are significantly different from each other (i.e. the C_α_-RMSD between them was 10.88 Å), and contain large water-soluble domains, they were subjected to a quality check by means of the 3D-Profile program [33], to help selection of the best one. Interestingly, for the native-like A-Bs3-Cs1-Ds1-Es6-Fs1-Gs1 eptamer, the 3D-Profile score was higher than that of the alternative eptamer (i.e. 438.0 vs 392.4, respectively), quite indicative of a more suitable packing of the seven subunits.

### Quaternary structure predictions of BRD

**Figure 9:**
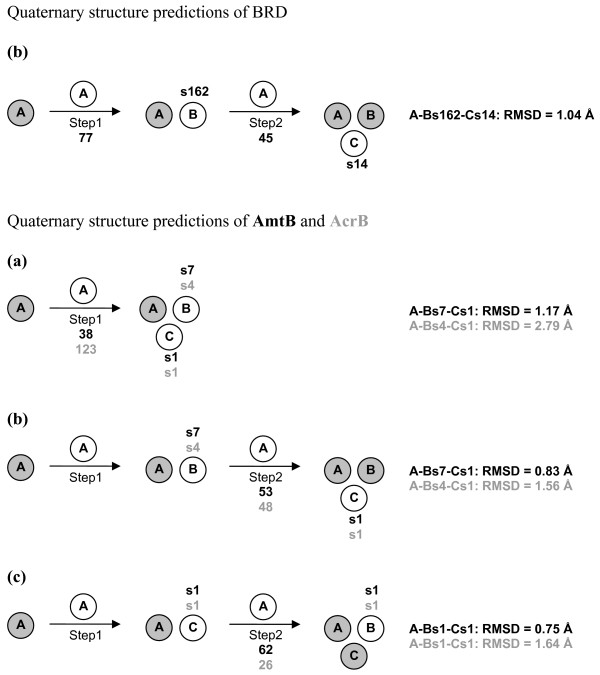
Prediction path for BRD (top) and for AmtB and AcrB (bottom).  The description of this figure is the same as that in Figure 6. For BRD, only one growing path has been pursued. In contrast, for quaternary structure predictions of AmtB and AcrB, the growing paths (a), (b) and (c) were probed. Black bold labels refer to AmtB, whereas gray bold labels refer to AcrB predictions.

For BRD, A vs A docking, followed by the application of the membrane topology filter, led to 77 solutions (Figure 9). These solutions essentially grouped in two clusters. One of these two clusters showed a better MemTop index than the other cluster (i.e. 0.537 vs 0.732, respectively) and was, thus, used to select the best scored solution, leading to the A-Bs162 dimer. A vs A-Bs162 docking led to 45 solutions, grouped in seven clusters. The solution selected based upon the criteria described above, s14, led to the A-Bs162-Cs14 trimer, characterized by a C_α_-RMSD of 1.04 Å from the native trimer (Table 1 and Figures 5b, 5c and 9 top). This is a striking result, considering also that the lipid molecules, which are present at the interfaces between the monomers in the crystal structure of the trimer, were completely neglected in docking simulations. Furthermore, the predicted trimer is made of identical monomers, whereas, in the crystal structure of the oligomer, slight differences exist between the monomers, concerning their internal coordinates and the main-chain length.

We probed also a different crystal structure of BRD, 1KME [34]. This structure differs from the one extracted from the native oligomer both in the backbone (i.e. 1.0 Å C_α_-RMSD in the 5–231 sequence, and 1.5 Å C_α_-RMSD in the intracellular and extracellular domains) and side-chain conformations. Following exactly the same path and approach as those used for the monomer extracted from the native trimer, a trimer was achieved characterized by a C_α_-RMSD of 4.0 Å from the native oligomer. It is worth noting that such C_α_-RMSD value comprises also the structural differences between the monomers in the native and predicted trimers.

### Quaternary structure predictions of the set of membrane proteins for which the asymmetric unit did not correspond to the biological unit

The subset of membrane proteins, KcsA, MscL and MscS and BRD, for which the asymmetric units corresponded to the biological unit, were employed for setting the prediction protocol. Benchmarks were then extended to a number of homo-oligomers, for which the asymmetric unit did not contain the biologic unit that was, hence, obtained by symmetry operations. These oligomers included: the tetrameric AQP1, GlpF and KirBac1, the trimeric AmtB and AcrB, as well as the dimeric BtuCD.

**Figure 10:**
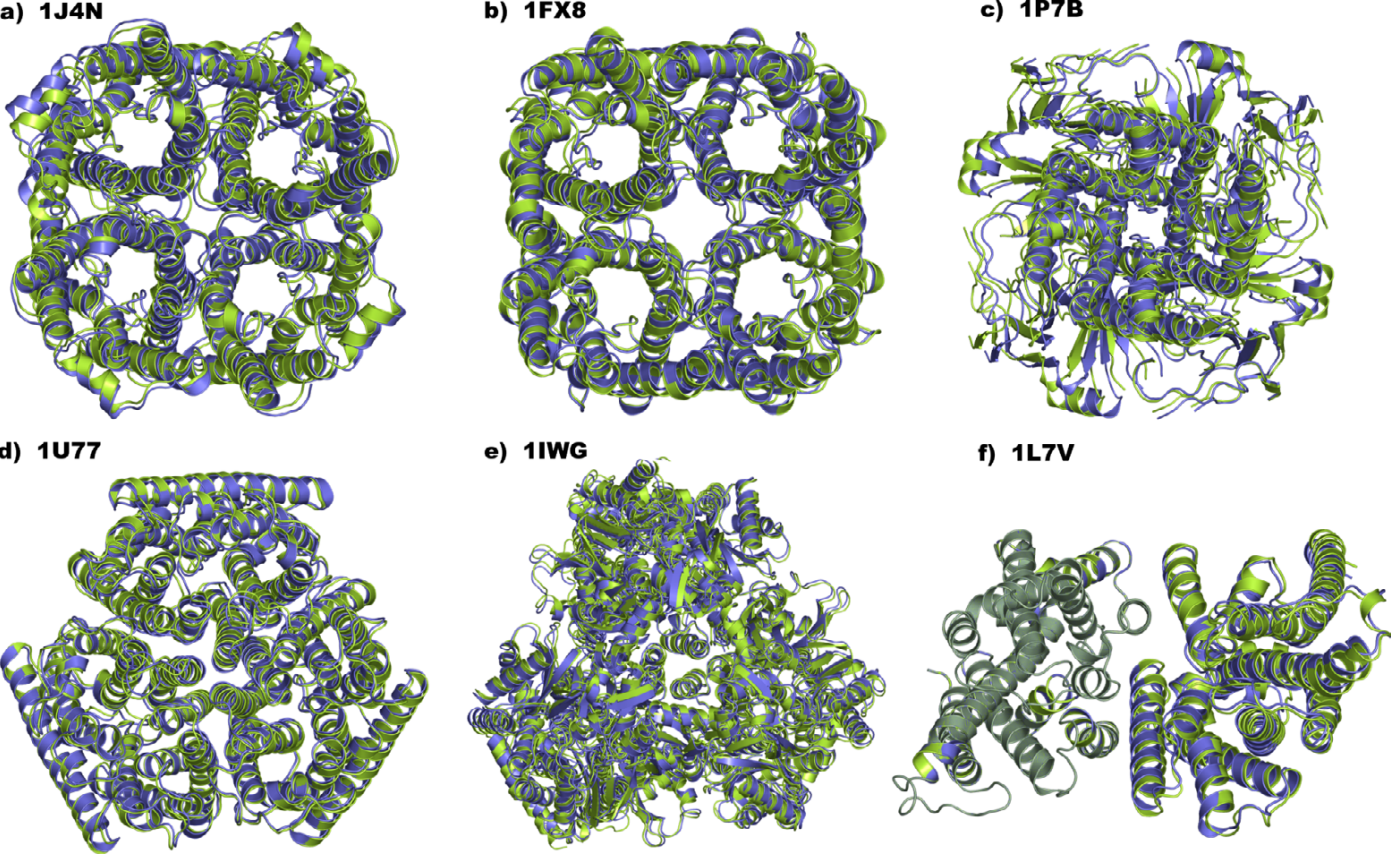
Superimposition between native (green color) and the best native-like (violet color) oligomeric structures of: (a) AQP1 (PDB code: 1J4N; C_α_-RMSD = 1.31 Å; best predicted tetramer: A-Bs2-Cs1-Ds1), (b) GlpF (PDB code: 1FX8; C_α_-RMSD = 1.11 Å; best predicted tetramer: A-Bs2-Cs1-Ds1), (c) KirBac1 (PDB code: 1P7B; C_α_-RMSD = 1.47 Å; best predicted tetramer: A-Bs3-Cs1-Ds3), (d) AmtB (PDB code: 1U77; C_α_-RMSD = 0.75 Å; best predicted trimer: A-Bs1-Cs1), (e) AcrB (PDB code: 1IGW; C_α_-RMSD = 1.56 Å; best predicted trimer: A-Bs4-Cs1), and (f) BtuCD (PDB code: 1L7V; C_α_-RMSD = 0.63 Å). For BtuCD, the native-like structure shown in this figure, i.e. the A-Bs1678 dimer, has been achieved through a dipole moment-based reorientation approach. The oligomers are seen from the extracellular side in a direction perpendicular to the putative membrane surface.

In detail, for the tetrameric AQP1, GlpF and KirBac1, the same growing paths as those employed for quaternary structure predictions of KcsA were probed. Similarly to KcsA, for these proteins, the two-step growing paths (a), (b) and (c) always resulted into native-like tetramers. In detail, for AQP1, the predicted tetramers (i.e. A-Bs2-Cs1-Ds1, A-Bs1-Cs2-Ds1 and A-Bs2-Cs4-Ds1) showed, respectively, C_α_-RMSDs equal to 1.31 Å, 1.71 Å and 1.37 Å from the native oligomer (Table 1 and Figure 10a). For GlpF, the predicted tetramers (i.e. A-Bs2-Cs1-Ds1, A-Bs1-Cs2-Ds1 and A-Bs2-Cs2-Ds1) showed, respectively, C_α_-RMSDs equal to 1.11 Å, 1.16 Å, and 1.39 Å from the native oligomer (Table 1 and Figure 10b). Finally, for KirBac1, the predicted tetramers (i.e. A-Bs3-Cs1-Ds1, A-Bs3-Cs1-Ds3 and A-Bs2-Cs1-Ds1) showed, respectively, C_α_-RMSDs equal to 1.53 Å, 1.47 Å and 1.8 Å from the native oligomer (Table 1 and Figure 10c). It is worth noting that, for all the predicted structures, the docking solutions contributing to each tetramer fell among the top 3 solutions out of 4000 in the output list. Furthermore, similarly to KcsA, for AQP1 and GlpF, the growing path, which employs a trimer as a target at step 2 (i.e. path (a)), produced the best native-like tetramer (Table 1).

Differently from BRD, for AmtB and AcrB, the MemTop indices of the two most populated clusters from the first docking step were similar. This made it possible to achieve trimers following either path (a) or (b) or (c) (Figure 9 bottom). In detail, for AmtB, A vs A docking led to 38 solutions essentially grouped in two equi-populated clusters showing also good and similar MemTop indices (i.e. 0.317 and 0.319). The best scored solutions from these two clusters, s1 and s7, were likely to form the C and B subunits, respectively. The A-Bs7-Cs1 trimer was characterized by a C_α_-RMSD of 1.17 Å from the native trimer. A vs A-Bs7 and A vs A-Cs1 docking, led to 53 and 62 reliable solutions, respectively, which essentially grouped in one cluster. The best solution from this cluster, s1, in both cases, led to the A-Bs7-Cs1 and A-Bs1-Cs1 native-like trimers characterized, respectively, by C_α_-RMSDs of 0.83 Å and 0.75 Å from the native trimer (Table 1 and Figures 9 and 10d). For AcrB, A vs A docking led to 123 solutions, essentially grouped in two clusters showing low and similar MemTop indices (i.e. 0.220 and 0.135). The best scored solutions from these two clusters led to the A-Bs4-Cs1 trimer showing a C_α_-RMSD equal to 2.78 Å from the native trimer. A vs A-Bs4 and A vs A-Cs1 docking, led to 48 and 26 reliable solutions, respectively, which essentially grouped in one cluster. The best solution from this cluster, s1 in both cases, led to the A-Bs4-Cs1 and A-Bs1-Cs1 native-like trimers characterized, respectively, by C_α_-RMSDs of 1.56 Å and 1.64 Å from the native trimer (Table 1 and Figures 9 and 10e).

For BtuCD, whose membrane-spanning domain is a dimer, A vs A docking led to 47 solutions distributed in 11 small clusters. Since the cluster population in this case is meaningless, we considered only the cluster characterized by the best MemTop index (i.e.0.384). The lowest scored solution from this cluster, s178, led to a dimer characterized by a C_α_-RMSD of 0.63 Å from the native complex (Table 1 and Figure 10f). These results were achieved following a dipole moment-based approach for subunit reorientation (see the Experimental Procedure section). The membrane topology-based reorientation approach, led to 48 reliable solutions, 30 of which were divided into 7 clusters, whereas the remaining 18 couldn't be clusterized. The 25 unique solutions contained the native-like dimer, characterized by one of the best MemTop index and a C_α_-RMSD of 0.48 Å from the native complex. However, in this case, retrieving the native-like solution couldn't be unequivocally done, as it didn't hold either the best ZDOCK score or the best MemTop index.

## Discussion

Following benchmarks, we have defined an effective computational protocol to predict the supramolecular structure of integral α-helical TM proteins, starting from the monomer. The approach was first tested on the trimeric BRD, tetrameric KcsA, pentameric MscL and eptameric MscS. These systems were selected following an accurate database search for high resolution structures of oligomeric membrane proteins, significantly different from each other and whose asymmetric crystallographic unit contains the biological unit. Docking samplings on these proteins were instrumental in setting the structure prediction protocol. Benchmarks were then extended to a number of homo-oligomers, for which the asymmetric unit did not contain the biologic unit that was, hence, obtained by symmetry operations. These oligomers included: the dimeric BtuCD, the trimeric AmtB and AcrB, and the tetrameric AQP1, GlpF and KirBac1.

For all the considered systems, native-like quaternary structures were achieved regardless of the resolution of the structural models and the extensions of the TM and water-exposed domains (Figures 2, 3, 4, 5, 6, 7, 8, 9, 10 and Table 1), thus, proving the effectiveness of the prediction approach. The latter consists in a number of dense docking samplings, starting from the docking of two identical copies of a given monomer. Each docking run is followed by membrane topology filtering and cluster analysis. Thus, prediction of the native oligomer is accomplished by a number of progressive growing steps, each made of one docking run, filtering and cluster analysis (Figure 1).

The only requirement with this approach is the structural model of the monomer that must hold the proper membrane topology. The correct membrane topology of the monomer, which in the first docking run of each growing path is employed in two identical copies (one for the target and the other for the probe), is, indeed, necessary for the membrane topology filter to work properly. This filter generally discards more than 94% of the total solutions provided by each docking run (i.e. 4000 solutions), thus allowing for an easy individuation of the clusters holding the native-like solutions. These clusters are, in fact, the one or two most populated, and hold also the best membrane topology indices, i.e a MemTop index close to zero. For almost all the docking runs, the best hit from the selected clusters fall within the first 10 out of the 4000 output solutions provided by the docking algorithm.

A significant number of trials on the four highly different systems BRD, KcsA, MscL and MscS allowed us to define the best criteria for selecting the proper docking solutions at each growing step and the best growing path. These criteria proved validity in quaternary structure predictions of the second set of membrane proteins.

Selection of the proper solution/s at each growing step should be based on the docking score as well as on the population and membrane topology of the solution clusters. In detail, if the realistic solutions group essentially in two clusters characterized also by similar and significantly low MemTop indices, the best scored solutions from both clusters should be selected to grow the oligomer. In contrast, if the two most populated clusters have significantly different MemTop indices, only the one showing the lowest index must be chosen for extracting the best scored solution. Finally, if one cluster is significantly most populated than the others, only that cluster must be considered for extracting the best scored solution.

As for the growing paths, in general, those should be pursued, which employ a dimeric target in the second step (Figures 6, 7, 8, 9). For cyclic oligomers, our approach is able to predict the oligomeric order of the functional unit, since completion of the cycle determines the end of the growth. Once established the oligomeric order, for systems like KcsA, AQP1 and GlpF, which are characterized by an even number of monomers, a growing path, which employs a trimer as a target at step 2, is worth attempting. In fact, in our benchmarks, this path led to the best tetramer, in terms of C_α_-RMSD from the native structure (Table 1, path (a)). Based on these results, we infer that, for even order oligomers, growing paths characterized by the employment of a trimeric target at step 2 may be preferred, whereas, for odd order oligomers, the preferential growing paths are those employing a dimeric target at step 2. Interestingly, both these paths share the characteristic that, in the final step, only one monomer is needed to complete the oligomer, i.e. to close the cycle. For odd order oligomers, this would imply pursuing two parallel growing paths, finally leading to two predicted quaternary structures. When the two predicted oligomers differ significantly from each other, quality checks are needed to select the final structure. In our study, this was the case of the MscS oligomer (Figure 8b and 8c). In fact, the two parallel growing paths passing through a dimeric target led to two significantly different oligomers (i.e. the C_α_-RMSD between them was 10.88 Å). In this case, given the significant extension of the water-exposed domains compared to the TM ones, the 3D-Profile score proved effectiveness in individuating the native-like oligomer.

For non-cyclic oligomers, the growth of the supramolecular assembly must stop when no more realistic solutions can be found. Low resolution information from Atomic Force Microscopy (AFM) or cryo-electron microscopy, if available, should be combined with the results of docking analysis. We couldn't do benchmarks on non-cyclic oligomers characterized by oligomeric orders higher than two or three due to the lack of high resolution structures for such systems. As an example, for BRD, we stopped the growth at the trimer, since the native structure, required for benchmarks, is available only for the trimer. However, we know from AFM images that BRD trimers organize in higher order oligomers [3]. Preliminary results of trimer vs trimer docking, reconstructed a BRD esamer similar to that inferred from AFM images (results not shown) [3]. We observed that the number of filtered solutions decreases significantly ongoing from monomer vs monomer to trimer vs trimer docking (i.e. from 77 to 15, respectively). This is in line with the fact that intra-trimer contacts should be stronger than the inter-trimer ones, which are mediated also by lipid molecules.

The effectiveness of predictions is independent of the content of water-exposed domains in the monomeric units, suggestive of the fundamental role of the TM domains in driving the supramolecular organization.

An important result is that predictions were excellent also for those systems, for which the asymmetric unit consists of the monomer and not of the biologic unit. These may be considered as unbound-unbound docking cases. Moreover, quaternary structure predictions can not be considered as bound-bound docking cases neither for those cases, in which the crystallographic asymmetric unit contains the biologic unit, and, hence, the monomer used for simulations was extracted from the X-ray structure of the complex. In fact, in none of the docking steps the situation is such that probe and target are the same components of the native complex. Moreover, for BRD, the predicted trimer is made of identical monomers, whereas, in the crystal structure of the oligomer, slight differences exist between the monomers, concerning their internal coordinates and the main-chain length. Moreover, the lipid molecules, which are present at the interfaces between the monomers in the crystal structure of the trimer, were completely neglected in our docking simulations. The employment of an "unbound" BRD structure (i.e. PDB code 1KME) [34], differing both in the backbone and side-conformations from the monomer extracted from the native trimer did not prevent the approach from leading to a correct quaternary structure.

## Conclusion

In conclusion, the benchmarks carried out in this study on ten homo-oligomeric membrane proteins with known quaternary structure validate the proposed structure prediction protocol that, for all the tested cases, led to native-like quaternary structures regardless of the resolution of the structural models.

Quaternary structure predictions will be, hence, extended to other integral membrane proteins, including GPCRs. An attempt in this respect has been already reported, though based on an early and different version of the computational protocol, proving usefulness in aiding the interpretation of biophysical data and the design of novel *in vitro *experiments[14,35]

## Methods

### Overview of the computational approach

Quaternary structure predictions were carried out through a stepwise approach consisting of a combination of rigid-body docking, membrane topology filtering, cluster analysis, and solution selection for the growth of the oligomer. Solution selection was essentially based on the docking score and the quality of the membrane topology (Figure 1).

Rigid body docking was carried out by means of the program ZDOCK 2.1, which utilizes the Pairwise Shape Complementarity (PSC) scoring function, neglecting desolvation [36].

For each protein system, the first docking run consisted of docking two identical copies of the crystal structure of monomer A, keeping one monomer fixed (i.e. target) and allowing the other copy of the monomer to move around the target (i.e. probe). For the membrane topology filter to work properly, the two identical copies of the starting monomer must have the appropriate orientation with respect to the putative membrane. This is due to the fact that ZDOCK expresses its docking solutions in terms of a x,y,z-translation and a RzRxRz-rotation of the probe. If both target and probe are properly oriented in a membrane parallel to the XY plane, the translation along the z-axis can be considered as an offset out of the membrane and the Rx component of the rotation as a deviation from the original orientation in the membrane. The membrane topology filter, indeed, discards all the solutions characterized by a deviation angle from the original z-axis, i.e. tilt angle, and a displacement of the geometrical center along the z-axis, i.e. z-offset, above defined threshold values. For the tilt angle and the z-offset, thresholds of 0.4 radians and 6.0 Å were, respectively, employed. The check of the correct orientation and the eventual reorientation of the monomer are automatically carried out in the following way. Membrane topology predictions allow for the definition of two sets of C_α_-atoms, which putatively lie at the two lipid/water interfaces of the membrane. The planes that fit at best these C_α_-atoms are found by means of a multiple linear regression approach. If these planes are parallel to the xy plane and, hence, perpendicular to the z-axis, the orientation of the monomer is considered good and no reorientation is needed. In contrast, if the monomer needs a reorientation, the rotations necessary to bring the two planes parallel to the xy plane are computed and then an average between the two rotations is employed to reorient the whole monomer. A number of membrane topology predictors were probed [37]. Overall, the PRODIV-TMHMM_0.91 predictor allowed for the most effective subunit reorientation. A reorientation was needed only for KcsA, BtuCD and the two different monomers of BRD (i.e. the one extracted from the trimer, PDB code 1BRR, and the monomeric structure encoded as 1KME). For KcsA and BtuCD we probed also an approach consisting in orienting the native oligomeric structure so that the dipole moment of the oligomer was parallel to the z-axis.

The codes for monomer reorientation and membrane topology filtering have been included in our FIPD software for the analyses of ZDOCK outputs [see Additional file 1][40].

For each set of docking simulations aimed at reaching the final oligomerization state of the protein, the runs that succeeded the first one were carried out by using the original monomer A as a probe and the intermediate oligomer as a target. For each of these runs, neither the probe monomer nor the target oligomer require a reorientation, since the former molecule is the same as that used in the first step, whereas the oligomeric target inherits the orientation of the original target.

Docking samplings were carried out by using a 128 × 128 × 128 point grid with a spacing of 1.2 Å and a rotational sampling interval of 6°, i.e. dense sampling. From each docking run, the first best 4000 solutions, according to the ZDOCK score, were retained. These solutions were subjected to the "membrane topology" filter described above. The filtered solutions from each run were merged with the target protein, leading to an equivalent number of oligomers that were subjected to cluster analysis. Clustering was carried out by using an algorithm from Dr. M. Schaefer (Michael Schaefer, Syngenta Crop Protection AG, unpublished work). The algorithm first calculates the Cα-RMSD for each superimposed pair of dimers/oligomers and then it computes the number of neighbors for each dimer/oligomer, using a threshold Cα-RMSD. The dimer/oligomer with the highest number of neighbors is considered as the center of the first cluster. All the neighbors of this configuration are removed from the ensemble of configurations to be counted only once. The center of the second cluster is then determined in the same way as for the first cluster, and this procedure is repeated until each structure is assigned to a cluster. The Cα-RMSD threshold value for each superimposed pair of dimers/oligomers was set equal to 3.0 Å. All the amino acid residues in the dimer/oligomer were included in C_α_-RMSD calculations.

To identify the cluster of solutions with the best membrane topology, i.e. with the lowest values of both tilt angle and z-offset, we defined the MemTop index, MemTop=〈tiltnor〉2+〈Zoffnor〉2
 MathType@MTEF@5@5@+=feaafiart1ev1aaatCvAUfKttLearuWrP9MDH5MBPbIqV92AaeXatLxBI9gBaebbnrfifHhDYfgasaacH8akY=wiFfYdH8Gipec8Eeeu0xXdbba9frFj0=OqFfea0dXdd9vqai=hGuQ8kuc9pgc9s8qqaq=dirpe0xb9q8qiLsFr0=vr0=vr0dc8meaabaqaciaacaGaaeqabaqabeGadaaakeaacqWGnbqtcqWGLbqzcqWGTbqBcqWGubavcqWGVbWBcqWGWbaCcqGH9aqpdaGcaaqaamaaamqabaGaemiDaqNaemyAaKMaemiBaWMaemiDaq3aaSbaaSqaaiabd6gaUjabd+gaVjabdkhaYbqabaaakiaawMYicaGLQmcadaahaaWcbeqaaiabikdaYaaakiabgUcaRmaaamqabaGaemOwaO1aaSbaaSqaaiabd+gaVjabdAgaMjabdAgaMjabd6gaUjabd+gaVjabdkhaYbqabaaakiaawMYicaGLQmcadaahaaWcbeqaaiabikdaYaaaaeqaaaaa@5032@, were <*tilt*_*nor*_> and <*Z*_*offnor*_> are, respectively, the normalized tilt angle and the z-offset averaged over all the members of a given cluster. Normalization of each tilt angle and z-offset value was carried out by dividing each value for the respective cutoff value, i.e. 0.4 radians, for the tilt angle, and 6.0 Å, for the z-offset. The optimal value for such index is zero.

The quality of some of the predicted oligomeric structures of MscS was estimated by means of the 3D-Profile program [33] within the Quanta 2000 package.

Molecular visualizations were done by means of the VMD v1.8.2 program [38].

As indicative values, a monomer vs monomer and monomer vs esamer docking for MscS required 87 hours and 147 hours, respectively, of CPU time on a 2.2 GHz Opteron with 2 GB RAM.

## Authors' contributions

DC carried out docking simulations and analyses. MS wrote the codes to allow effective analyses of the docking outputs. FF conceived and coordinated the study and wrote the manuscript. All authors read and approved the final manuscript.

## Supplementary Material

Additional File 1 fipd. Archive file containing two files: (a) the FIPD software compiled for Linux machines (i.e. fipd_i386.x), and (b) the relative documentation (README). Such software allows for both monomer reorientation and ZDock solution filtering.This software can be also downloaded from the authors' WEB site [40]. Click here for file
